# Supplementary material for: Targeting the Retinoblastoma/E2F repressive complex by CDK4/6 inhibitors amplifies oncolytic potency of an oncolytic adenovirus
Source: Nat Commun. 2022 Aug 10;13:4689. doi: 10.1038/s41467-022-32087-5 (PMC9365808; doi:10.1038/s41467-022-32087-5)
Supplement: Supplementary file 1 — Supplementary Information [file 41467_2022_32087_MOESM1_ESM.pdf]

Targeting the Retinoblastoma/E2F repressive complex by CDK4/6 inhibitors amplifies oncolytic potency of an oncolytic adenovirus

Author List:

Jana Koch[1,6,†], Sebastian J. Schober[2,†], Sruthi V. Hindupur[1,†], Caroline Schöning[2], Florian G. Klein[1], Klaus Mantwill[1], Maximilian Ehrenfeld[1], Ulrike Schillinger[1,], Timmy Hohnecker[1], Pan Qi[1,7], Katja Steiger[3], Michaela Aichler[4], Jürgen E. Gschwend[1], Roman Nawroth[1,*], Per Sonne Holm[1,5,*]

Supplementary information

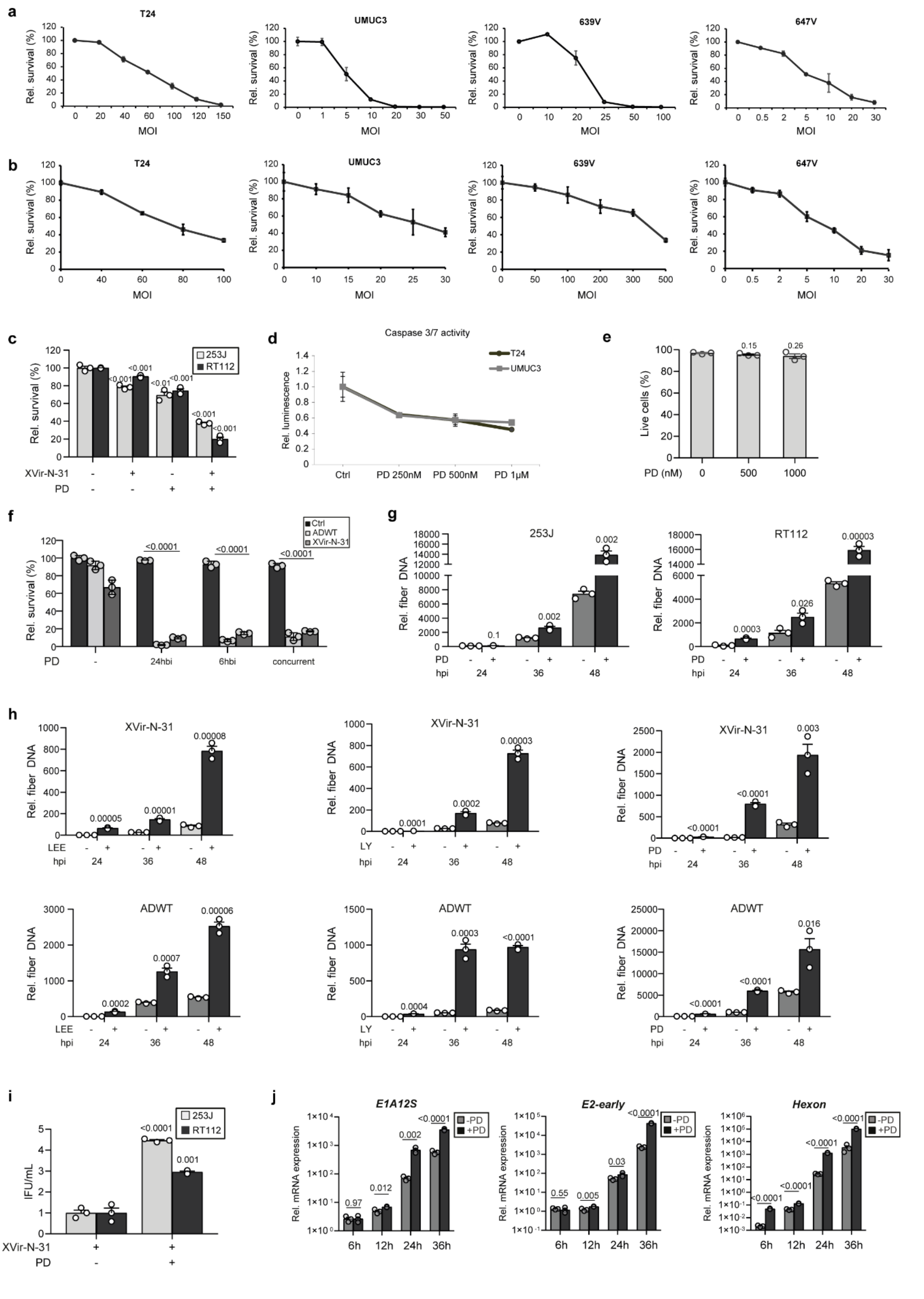

Supplementary Figure-1: Oncolytic virotherapy is enhanced in combination with CDK4/6 inhibitors in bladder cancer (a,b) Cell proliferation analyses in T24, UMUC-3, 639V and 647V cells infected with increasing MOIs of ADWT and XVir-N-31. **(c)** Cell proliferation analyses in RT112 and 253J cells. Cells were pre-treated for 24hrs with Palbociclib (RT112 2000nM, 253J 100nM) and infected with XVir-N-31 (RT112 MOI 400, 253J MOI 20). Cell viability was assessed at 4dpi. Data is shown as percentage of surviving cells (mean ± SE) relative

to non-infected, untreated control. p<0.05. (d) Caspase 3/7 activity was assessed in T24 and UMUC-3 cells treated with increasing concentrations of Palbociclib for 24 hrs. (n=3, mean ± SE) (e) Live cell counting was performed by Trypan blue staining in T24 cells treated with indicated concentrations of Palbociclib for 24 hrs. Data is shown as live cell percentage among total cells (mean ± SE). (f) T24 cells were treated with Palbociclib for indicated time points and infected with ADWT (MOI 150) and XVir-N-31 (MOI 80). Cell viability was assessed at 4dpi. Data is shown as the percentage of surviving cells (n=3, mean ± SE) relative to non-infected, untreated control. p<0.05. (g) Viral genome replication was assessed by qPCR for fiber DNA in RT112 and 253J cells pre-treated for 24hrs with Palbociclib (RT112 2000nM, 253J 100nM) and infected with XVir-N-31 (RT112 MOI 450, 253J MOI 25). Data is represented as relative fiber DNA at indicated time points compared to fiber DNA at 4hpi as baseline. (n=3, mean±SD). **(h)** T24 cells were infected with XVir-N-31 or ADWT (MOI 50) and where indicated, pre-treated with CDK4/6 inhibitors (LY2835219 and PD-0332991 500nM, LEE011 5000nM). Viral genome replication was analysed at indicated time points. (n=3, mean±SD). **(i)** Viral particle formation is assessed in cells pre-treated with Palbociclib (RT112 2000nM, 253J 100nM) and infected with XVir-N-31 (RT112 MOI 450, 253J MOI 25). The increase in viral titre upon CDK4/6 inhibition is given in infectious units/ml (IFU/ml). (n=3, mean ± SE) **(j)** Viral gene expression was assessed at indicated time points in T24 cells that were pre-treated with 500nM Palbociclib and infected with XVir-N-31 (MOI 50). The data shown represents the mRNA expression relative to reference gene beta-Actin. (n=3, mean±SD). SD: standard deviation, SE: standard error, hpi/dpi: hours/days post infection, MOI: multiplicity of infection. The statistical significance was determined by two-sided Student's t-test. n: number of biologically independent samples. Source data are provided as a Source Data file.

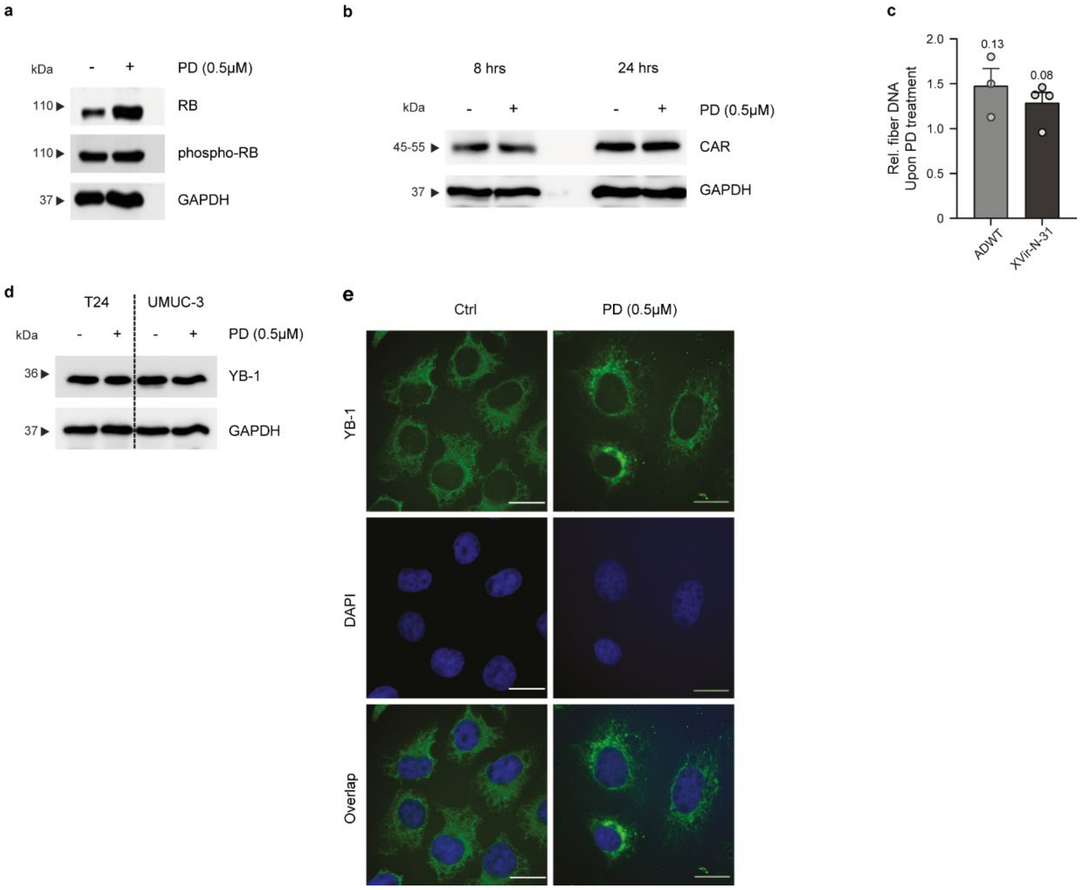

Supplementary Figure-2: CDK4/6 inhibition does not affect infectivity of cells (a) Protein expression of RB and phosphor-RB were analysed 24 hrs after Palbociclib treatment in Hek293 cells by western blotting. (b) UMUC3 cells were treated for 8 or 24 hours with Palbociclib and CAR protein expression levels were analysed by western blotting. (c) Fiber-DNA was detected by qPCR 4 hours after infection with ADWT or XVir-N-31 (MOI 50) in UMUC3 cells (n=4, mean±SD). (d) Protein expression of YB1 was analysed 24 hrs after Palbociclib treatment in indicated cell lines by western blotting. (e) Representative immunofluorescence images depicting staining for YB-1 protein in UMUC3 cells treated with Palbociclib for 24hrs. Scale bars represent 100µM. SD: standard deviation. The statistical significance was determined by two-sided Student's t-test.). One representative blot is shown of three independent experiments. n: number of biologically independent samples. Source data are provided as a Source Data file.

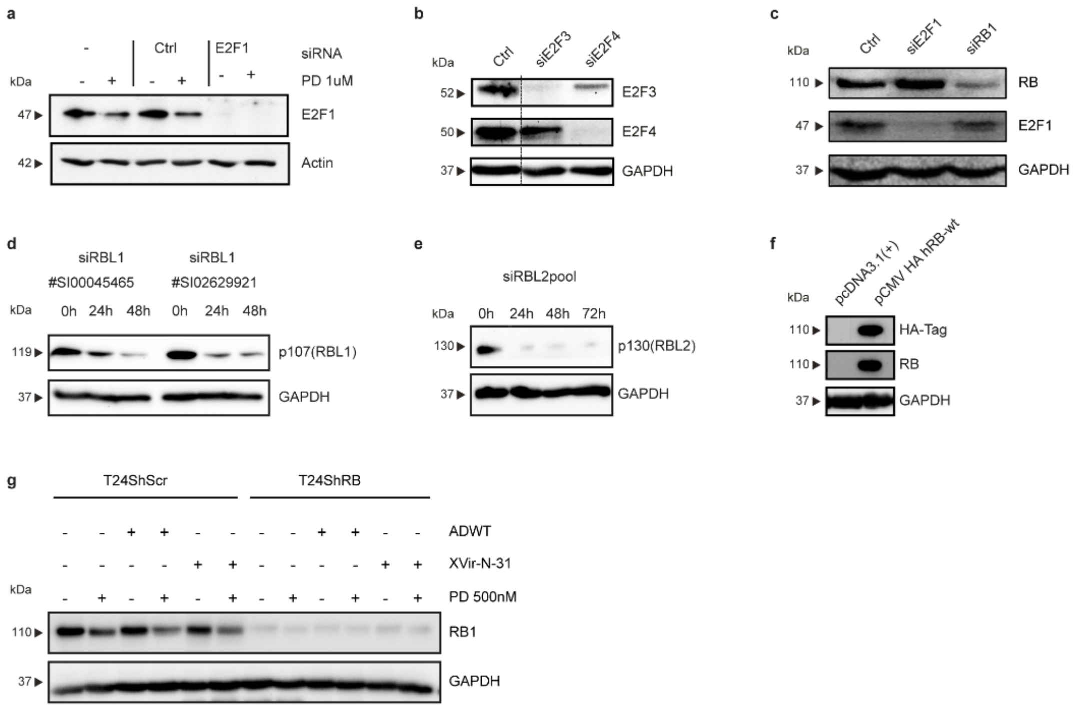

Supplementary figure-3: E2F and RB family proteins are efficiently downregulated using siRNAs. Knockdown and overexpressions were confirmed at 24 hrs after transfection by western blotting in (a) T24 cells transfected with siE2F1 pool (b) T24 cells transfected with siE2F3, 4 siPools (c) SK-N-MC cells transfected with siE2F1 and siRB (d) SK-N-MC cells transfected with siRBL1 (p107) (e) SK-N-MC cells transfected with siRBL2 (p130) pool (f) Saos-2 cells transfected with pCMV HA hRB-wt plasmid. (g) T24ShScr andT24ShRB cells were treated with 500nM Palbociclib, and infected with either ADWT or XVir-N-31 (MOI 50). RB protein expression was detected by western blotting. One representative blot is shown of three independent experiments. Source data are provided as a Source Data file.

a

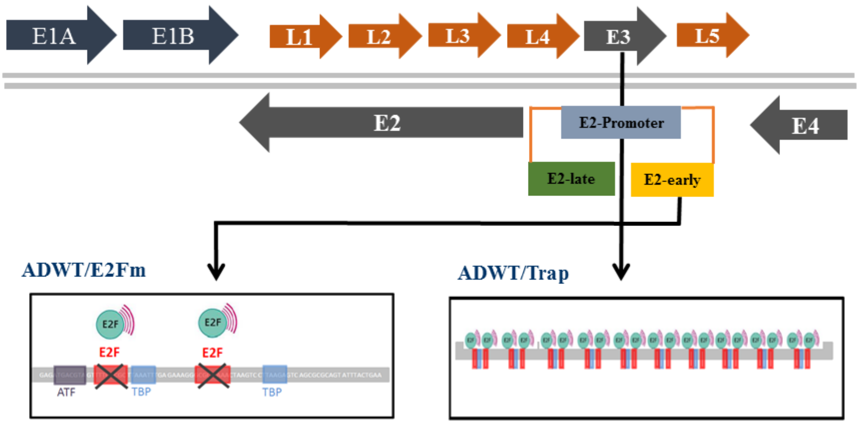

b

```
 PmeI   BamHI         E2F Bind I                  E2F Bind II              E2F Bind I
GTTTAAACGGATCCGgactagtttcgcgccctttctcaaatttaagcgcgaaaactacgtcatggactagtttcgcgcccttt

           E2F Bind II                 E2F Bind I                 E2F Bind II
ctcaaatttaagcgcgaaaactacgtcatggactagtttcgcgccctttctcaaatttaagcgcgaaaactacgtcatggact

 E2F Bind II                E2F Bind I                 E2F Bind II              E2F Bind I
agtttcgcgccctttctcaaatttaagcgcgaaaactacgtcatggactagtttcgcgccctttctcaaatttaagcgcgaaa

                       E2F Bind I                E2F Bind II              E2F Bind I
actacgtcatAGATCCGgactagtttcgcgccctttctcaaatttaagcgcgaaaactacgtcatggactagtttcgcgccct

             E2F Bind II                E2F Bind I                 E2F Bind II
ttctcaaatttaagcgcgaaaactacgtcatggactagtttcgcgccctttctcaaatttaagcgcgaaaactacgtcatgga

  E2F Bind II                E2F Bind I                 E2F Bind II             E2F Bind I
ctagtttcgcgccctttctcaaatttaagcgcgaaaactacgtcatggactagtttcgcgccctttctcaaatttaagcgcga

aaactacgtcatAGATCTccagcgCTCGAGGTTTAAAC
           BglII          XhoI   PmeI
```

Supplementary Figure-4: Cloning strategy of E2F-modifications in adenoviral vectors. (a) Structure of ADWT/E2Fm and ADWT/Trap (b) Cloning of the Trap-Sequence into the genome of ADWT.

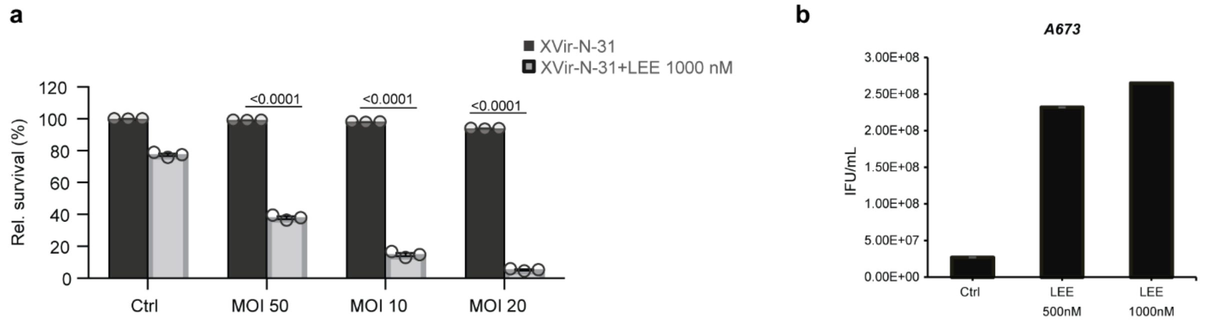

Supplementary Figure-5: Oncolytic virotherapy is enhanced in combination with CDK4/6 inhibitors in Sarcoma. (a) Cell proliferation analyses and viral particle formation of XVir-N-31-infected A673 Ewing sarcoma cell line in combination with CDK4/6 inhibition. Cell proliferation analyses in A673 cells. Cells were pre-treated for 24hrs with Ribociclib (LEE) and infected with XVir-N-31 at indicated MOI. Cell viability was assessed at 4dpi. Data is shown as percentage of surviving cells (n=3, mean ± SE) relative to non-infected, untreated control. (b) Quantification of viral particles was performed by a hexon titer test and expressed as infectious units per milliliter (IFU/ml). (n=3, mean ± SE). The statistical significance was determined by two-sided Student's t-test. n: number of biologically independent samples. Source data are provided as a Source Data file.

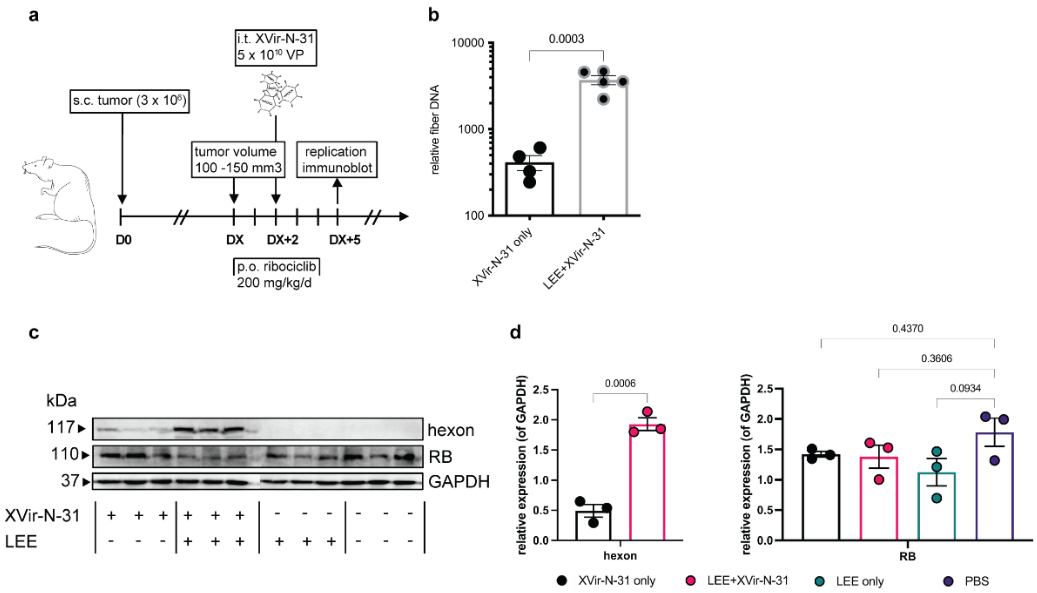

Supplementary Figure-6: Viral genome replication and hexon/RB levels in combination and monotherapy in 2nd xenograft mouse model. (a) Experimental design of the *in vivo* study (Rag2$^{-/-}\gamma c^{-/-}$mice). Ewing sarcoma cell line TC32 was implanted s.c. (3 x 10^6 cells). VP = viral particles. (**b)** Viral genome replication within the tumour is assessed by qPCR to amplify viral fiber DNA in three independent tumours in monotherapy (XVir-N-31 only) and in combination therapy (LEE+XVir-N-31) at day X+5 (DX+5). Error bars SEM. **(c)** Immunoblot analysis: each lane presents one explanted tumour and **(d)** depicts the densitometric quantification of the immunoblots per group and protein, as indicated (done with ImageJ 1.53k). Unpaired, two-tailed student's t-test was used to compare hexon levels and Tukey's multiple comparison in combination with ordinary one-way ANOVA was used for statistical analysis of RB protein levels (GraphPad Prism). Source data are provided as a Source Data file.

Supplementary Table-1: Selected pairwise comparisons of in vivo data.

Table-1a. Cross-sectional analysis day 12-21.

Selected pairwise comparisons.

p-value adjustment/Adj):Holm

Group 1	Group 2	Difference	P-value	P-value Adj	Wilcox P-value	Wilcox P-value Adj
LEE	PBS	-257.110 [-452.801;-61.420]	0.0007	0.0015	0.0173	0.042
XVir only	PBS	-518.429 [-707.660;-329.199]	<0.0001	<0.0001	0.0025	0.0126
combo	PBS	-684.618 [-873.848;-495.387]	<0.0001	<0.0001	0.0025	0.0126
XVir only	LEE	-261.319 [-441.115;-81.522]	0.0002	0.0006	0.014	0.042
combo	LEE	-427.507 [-607.304;-247.711]	<0.0001	<0.0001	0.0012	0.007
combo	XVir only	-166.189 [-338.932;6.554]	0.0134	0.0134	0.0262	0.042

Table-1b. Longitudinal analyses day 0-25.

Selected pairwise comparisons.

p-value adjustment/Adj):Holm

Largest	Smallest	Contrast	Df	P-value	P-value Adj
PBS	LEE	12.610 [3.156;22.065]	27.98	0.0108	0.0216
PBS	XVir only	29.459 [20.260;38.657]	28.63	<0.0001	<0.0001
PBS	combo	38.891 [29.750;48.031]	27.79	<0.0001	<0.0001
LEE	XVir only	16.848 [8.708;24.988]	23.12	0.0003	0.0008
LEE	combo	26.280 [18.203;34.357]	22.22	<0.0001	<0.0001
XVir only	combo	9.432 [1.659;17.206]	22.5	0.0196	0.0216

Supplementary methods

Supplementary Table -2: List of primers

Name	Forward primer	Reverse primer
Beta-actin	TAAGTAGGTGCAC AGTAGGTCTGA	AAAGTGCAAAGAA CACGGCTAAG
Beta-actin cDNA	CATGTACGTTGCT ATCCAGGC	CTCCTTAATGTCAC GCACGAT
E1A12S	CGACGAGGATGAA GTCCTGTGTCTG	CTCAGGATAGCAG GCGCCAT
E1A13S	TGTTTGTCTACAG TCCTGTGTCTG	CTCAGGATAGCAG GCGCCAT
E1A enhancer	TTTGGGCGTAAC CGAGTAAG	CAGCCAGTACCT CTTCGATC
E1B55K	CCTGGCCAGTGT TTGAGCAT	CCCGTTCAGGTTCA CCTTGG
E2-early	CCGTCATCTCTAC AGCCCAT	GGGCTTTGTCAGA GTCTTGC
E2F1	ACGCTATGAGACC TCACTGAA	TCCTGGGTCAACC CCTCAAG
E2-late	CTTCCTAGCGACT TTGTGCC	GTCAGAGTGGTAG GCAAGGT
E4ORF6	TCCCTCCCAACACA CAGAGT	GACAGGAAACCGTG TGGAAT
Hexon	TCCCTCCCAACAC ACAGAGT	GACAGGAAACCGT GTGGAAT
Fiber	AAGCTAGCCCTGC AAACATCA	CCCAAGCTACCAG TGGCAGTA
GAPDH	TGGCATGGACTGT GGTCATGAG	ACTGGCGTCTTCA CCACCATGG
RB	AGCAACCCTCCTA AACCACT	TGTTTGAGGTATC CATGCTATCA

Supplementary Table-3: List of antibodies used in western blotting

**Product**	**Catalog no.**	**Manufacturer**	**Dilution**
Beta-actin	A2066	Sigma-Aldrich Chemie GmbH	1:1000
CAR-D3W3G	16984	Cell Signaling Technology	1:1000
Cdc25A	sc-56264	Santa Cruz Biotechnology	1:1000
CDK2	2546	Cell Signaling Technology	1:1000
Chk1	sc-377231	Santa Cruz Biotechnology	1:2000
phospho Chk1 (Ser 317)	2344	Cell Signaling Technology	1:1000
Cyclin D1	2978	Cell Signaling Technology	1:1000
Cyclin E2	4132	Cell Signaling Technology	1:1000
E1A	sc-25	Santa Cruz Biotechnology	1:1000
E1B55k		Kindly provided by M. Dobbelstein, Göttingen University, Germany	1:100
E2A		Kindly provided by M. Dobbelstein, Göttingen University, Germany	1:100
E2F1	sc-251	Santa Cruz Biotechnology	1:1000
E2F2	ab138515	Abcam	1:1000
E2F3	MA1-25333	Thermo FisherScientific	1:1000
E2F4	MA5-11276	Thermo Fisher Scientific	1:1000
E2F5	sc-999	Santa Cruz Biotechnology	1:500
GAPDH	2118	Cell Signaling Technology	1:1000
Hexon	ABIN2686029	Antibodies online	1:1000
p107	ab209546	Abcam	1:1000
p130	ab76234	Abcam	1:1000
RB	554136	BD Biosciences	1:1000
phospho RB (Ser 780)	8180	Cell Signaling Technology	1:1000
YB1	ab12148	Abcam	1:500
