# Supplementary material for: Targeting the Retinoblastoma/E2F repressive complex by CDK4/6 inhibitors amplifies oncolytic potency of an oncolytic adenovirus
Source: Nat Commun. 2022 Aug 10;13:4689. doi: 10.1038/s41467-022-32087-5 (PMC9365808; doi:10.1038/s41467-022-32087-5)
Supplement: Supplementary file 3 — Reporting Summary [file 41467_2022_32087_MOESM3_ESM.pdf]

nature portfolio

Corresponding author(s): Roman Nawroth, Per-Sonne Holm

Last updated by author(s): Jun 8, 2022

Reporting Summary

Nature Portfolio wishes to improve the reproducibility of the work that we publish. This form provides structure for consistency and transparency in reporting. For further information on Nature Portfolio policies, see our Editorial Policies and the Editorial Policy Checklist.

Statistics

For all statistical analyses, confirm that the following items are present in the figure legend, table legend, main text, or Methods section.

n/a	Confirmed	
☐	☒	The exact sample size (n) for each experimental group/condition, given as a discrete number and unit of measurement
☐	☒	A statement on whether measurements were taken from distinct samples or whether the same sample was measured repeatedly
☐	☒	The statistical test(s) used AND whether they are one- or two-sided<br>*Only common tests should be described solely by name; describe more complex techniques in the Methods section.*
☐	☒	A description of all covariates tested
☐	☒	A description of any assumptions or corrections, such as tests of normality and adjustment for multiple comparisons
☐	☒	A full description of the statistical parameters including central tendency (e.g. means) or other basic estimates (e.g. regression coefficient) AND variation (e.g. standard deviation) or associated estimates of uncertainty (e.g. confidence intervals)
☐	☒	For null hypothesis testing, the test statistic (e.g. F, t, r) with confidence intervals, effect sizes, degrees of freedom and P value noted<br>*Give P values as exact values whenever suitable.*
☒	☐	For Bayesian analysis, information on the choice of priors and Markov chain Monte Carlo settings
☒	☐	For hierarchical and complex designs, identification of the appropriate level for tests and full reporting of outcomes
☒	☐	Estimates of effect sizes (e.g. Cohen's d, Pearson's r), indicating how they were calculated

Our web collection on statistics for biologists contains articles on many of the points above.

Software and code

Policy information about availability of computer code

Data collection	Immunoblots were acquired from ChemiDoc Imaging System with ImageLab Touch software version 2.4.0.03 (Bio-Rad). Images were acquired from Axio Vert.A1 microscope and Camera Axiocam ERc5s with ZEN 2012 (blue edition) software version 1.1.11. Luciferase activity was measured with Perkin Elmer 2030 workstation.ink (2018). Sulphorhodamine-B assays were acquired with Softmax Pro 7.1.2. qPCR data was acquired on a CFX96 Maestro 2.2 (5.2.008.0222) (Bio-Rad). Electron microscopy data was acquired with a Zeiss EM10CR transmission electron microscope at 60kV.
Data analysis	Statistical analysis was performed using GraphPad Prism 9.0.2(161) or Microsoft Excel 2019.

For manuscripts utilizing custom algorithms or software that are central to the research but not yet described in published literature, software must be made available to editors and reviewers. We strongly encourage code deposition in a community repository (e.g. GitHub). See the Nature Portfolio guidelines for submitting code & software for further information.

Data

Policy information about availability of data

All manuscripts must include a data availability statement. This statement should provide the following information, where applicable:

- Accession codes, unique identifiers, or web links for publicly available datasets
- A description of any restrictions on data availability
- For clinical datasets or third party data, please ensure that the statement adheres to our policy

All data are available in the manuscript, the supplemental information or the Source Data files.

Human research participants

Policy information about studies involving human research participants and Sex and Gender in Research.

Reporting on sex and gender	n/a
Population characteristics	n/a
Recruitment	n/a
Ethics oversight	n/a

Note that full information on the approval of the study protocol must also be provided in the manuscript.

Field-specific reporting

Please select the one below that is the best fit for your research. If you are not sure, read the appropriate sections before making your selection.

☒ Life sciences ☐ Behavioural & social sciences ☐ Ecological, evolutionary & environmental sciences

For a reference copy of the document with all sections, see nature.com/documents/nr-reporting-summary-flat.pdf

Life sciences study design

All studies must disclose on these points even when the disclosure is negative.

Sample size	For in vivo experiments, previous published results (Schober et al. 2020, Mantwill et al. 2013) were used to estimate the sample size for appropriate statistical testing. The exact n numbers, which were used, are indicated in respective figures and figure legends.For invitro experiments, no sample size calculations were performed. Sample sizes were determined to be adequate based on the magnitude and consistency of measurable differences between groups. The number of replicates or samples is indicated in each figure.
Data exclusions	In vivo experiments: Mice with ulcerating tumors, which had to be sacrificed before reaching the maximal permitted tumor burden, were excluded from survival analysis.
Replication	The reproducibility of the data was confirmed in multiple biological replicates as indicated in the figure legends.
Randomization	In vivo murine experiments: All mice were randomly allocated to treatment and control groups and were treated with regimens as indicated in the figures and figure legends.
Blinding	In vivo experiments were rated in a blinded manner by the senior investigators. Blinding after randomization was not applicable as the researchers applied therapeutic agents, measured tumor size, and performed further downstream analysis. For every experiment, several control samples were available and the applied strategy for data analysis was identical for every single sample.

Reporting for specific materials, systems and methods

We require information from authors about some types of materials, experimental systems and methods used in many studies. Here, indicate whether each material, system or method listed is relevant to your study. If you are not sure if a list item applies to your research, read the appropriate section before selecting a response.

Materials & experimental systems

n/a	Involved in the study
☐	☒ Antibodies
☐	☒ Eukaryotic cell lines
☒	☐ Palaeontology and archaeology
☐	☒ Animals and other organisms
☒	☐ Clinical data
☒	☐ Dual use research of concern

Methods

n/a	Involved in the study
☒	☐ ChIP-seq
☒	☐ Flow cytometry
☒	☐ MRI-based neuroimaging

Antibodies

Antibodies used

Primary antibodies:
Beta-actin (#A2066, Sigma-Aldrich, 1:1000), CAR-D3W3G (#16984, Cell Signaling Technology, 1:1000, Lot.no.1), Cdc25A (#sc-56264, Santa Cruz Biotechnology, 1:1000), CDK2 (2546, Cell Signaling Technology, 1:1000, Lot. No. 6), Chk1 (#sc-377231, Santa Cruz Biotechnology, 1:2000, Lot.no. I1013), phospho Chk1 (Ser 317) (#2344, Cell Signaling Technology, 1:1000, Lot.no.12), Cyclin D1 (#2978, Cell Signaling Technology, 1:1000, Lot.no.13), Cyclin E2 (#4132, Cell Signaling Technology, 1:1000, Lot.no.2), E1A (#sc-25, Santa Cruz Biotechnology, 1:1000, Lot.no. F0619), E1B55k and E2A (Kindly provided by M. Dobbelstein, Göttingen University, Germany, 1:100), E2F1 (#sc-251, Santa Cruz Biotechnology, 1:1000, Lot.no.B2916), E2F2 (#ab138515, Abcam, 1:1000, Lot.no. GR106430-7), E2F3 (#MA1-25333, Thermo Fisher Scientific, 1:1000, Lot.no. TK2672966E), E2F4 (#MA5-11276, Thermo Fisher Scientific, 1:1000, Lot.no. RD2177911), E2F5 (#sc-999, Santa Cruz Biotechnology, 1:500, Lot.no. K1714), GAPDH (#2118, Cell Signaling Technology, 1:1000, Lot.no.14), Hexon (#ABIN2686029, Antibodies online, 1:1000, Lot.no. AM0212), p107 (#ab209546, Abcam, 1:1000), p130 (#ab76234, Abcam, 1:1000, Lot.no. GR177624-4), RB (#554136, BD Biosciences, 1:1000, Lot.no.1229976), phospho RB (Ser 780) (#8180, Cell Signaling Technology, 1:1000,Lot.no. 7), YB1 western blotting (#ab12148, Abcam, 1:500, Lot.no. GR32520-1), YB-1 immunofluorescence (#ab76540, Abcam, 1:60)
Secondary antibodies:
Anti-rabbit IgG, HRP-linked antibody (#711-036, 152, Jackson Immuno Research, 1:10000, Lot.no. 148257), Anti-mouse IgG, HRP-linked antibody (#715-036, 150, Jackson Immuno Research, 1:10000, Lot.no. 143353), goat anti-rabbit IgG Alexa Fluor 488 (#A11008, Invitrogen, 1:10000, Lot.no. 2179202)

Validation

All the commercial antibodies have been validated by the manufacturers. Antibodies critical for the conclusions were validated by elimination of signals upon siRNA mediated knockdown experiments.

Eukaryotic cell lines

Policy information about cell lines and Sex and Gender in Research

Cell line source(s)

T24 (HTB-4TM) and Hek293 (CRL-1573), A673 (CRL-1598) cells were purchased from ATCC, VA, USA. SK-N-MC (ACC 203) and RT112 (ACC 418) cells were obtained from the German Collection of Microorganisms and Cell Cultures (DSMZ, Germany). TC32 was obtained from the Childhood Cancer Repository (CCR, Alex's Lemonade Stand Foundation, Children's Oncology Group, COG). UMUC-3, 639V and 647V were obtained from Professor WA Schulz, Düsseldorf, Germany. 253J were a kind gift from Professor G. Unteregger, Homburg, Germany.

Authentication

Authentification of cell lines was confirmed by genotyping analyzing short tandem repeats in 2016.

Mycoplasma contamination

Cell lines were tested for Mycoplasma contamination and found to be negative.

Commonly misidentified lines
(See ICLAC register)

We did not use misidentified cell lines in the manuscript

Animals and other research organisms

Policy information about studies involving animals; ARRIVE guidelines recommended for reporting animal research, and Sex and Gender in Research

Laboratory animals

Female NMRI-Foxn1 nu/nu mice (nude mice) were purchased from Charles River Laboratories and Rag2-/-γc-/- mice (BALB/c background, initially obtained from the Central Institute of Experimental Animals, Kawasaki, Japan) were bred and maintained in our animal facility (Center for Preclinical Research, School of Medicine, TUM) under specific pathogen-free (SPF) conditions in consideration of dark/light cycle, ambient temperature and humidity, according to the guidelines of the Federation of Laboratory Animal Science Association. Female mice between 10-20 weeks of age were used in experiments. All experiments with these mice were approved by the local regulatory authorities i.e. Regierung von Oberbayern (55.2-2532.Vet_02-17-225, 55.2-2532.Vet_02-15-102, 55.2-2532.Vet_02-20-165).

Wild animals

No wild animals were used in this study.

Reporting on sex

n/a

Field-collected samples	No field-collected samples were used in this study.
Ethics oversight	All animals were kept according to the guidelines of the Federation of Laboratory Animal Science Association and experiments were authorized by permission of the local regulatory authorities (Regierung von Oberbayern) in accordance to German Federal Law and institution guidelines (permission numbers: 55.2-2532.Vet_02-17-225, 55.2-2532.Vet_02-15-102, 55.2-2532.Vet_02-20-165).

Note that full information on the approval of the study protocol must also be provided in the manuscript.
